# Current Applications of Magnetic Nanomaterials for Extraction of Mycotoxins, Pesticides, and Pharmaceuticals in Food Commodities

**DOI:** 10.3390/molecules26144284

**Published:** 2021-07-15

**Authors:** Sarem Targuma, Patrick B. Njobeh, Patrick G. Ndungu

**Affiliations:** 1 Energy, Sensors and Multifunctional Nanomaterials Research Group, Department of Chemical Sciences, Doornfontein Campus, University of Johannesburg, Johannesburg 2028, South Africa; sarem.ter@gmail.com; 2 Department of Biotechnology and Food Technology, Doornfontein Campus, University of Johannesburg, Johannesburg 2028, South Africa; pnjobeh@uj.ac.za

**Keywords:** environment, food commodities, pollutants, magnetic nanomaterial, extraction

## Abstract

Environmental pollutants, such as mycotoxins, pesticides, and pharmaceuticals, are a group of contaminates that occur naturally, while others are produced from anthropogenic sources. With increased research on the adverse ecological and human health effects of these pollutants, there is an increasing need to regularly monitor their levels in food and the environment in order to ensure food safety and public health. The application of magnetic nanomaterials in the analyses of these pollutants could be promising and offers numerous advantages relative to conventional techniques. Due to their ability for the selective adsorption, and ease of separation as a result of magnetic susceptibility, surface modification, stability, cost-effectiveness, availability, and biodegradability, these unique magnetic nanomaterials exhibit great achievement in the improvement of the extraction of different analytes in food. On the other hand, conventional methods involve longer extraction procedures and utilize large quantities of environmentally unfriendly organic solvents. This review centers its attention on current applications of magnetic nanomaterials and their modifications in the extraction of pollutants in food commodities.

## 1. Introduction

Mycotoxins, pesticides, and pharmaceuticals are groups of pollutants that contaminate water, and food and feed samples, posing a health threat to consumers of the contaminated products. These pollutants or contaminants enter the environment through numerous pathways. Pharmaceutical compounds are often partially metabolized in humans and animals, and they are then excreted through urine or feces. Eventually, these compounds reach wastewater treatment plants where they are only partially removed, or transformed, and then released into receiving water bodies. Besides pharmaceuticals, wastewater treatment plants are typically unable to completely remove several classes of pollutants, such as personal care products, industrial run-offs or chemicals, micro/nano-plastics, and many others [1,2,3,4,5]. 

Other pathways for the movement of pollutants and contaminants into the environment include run-offs and wastewater from farms [6,7], storm water from urban and semi-urban areas [8], hospital discharges [2,5,9], and other non-point sources. Once these pollutants enter surface or ground water resources, they can eventually contaminate drinking and agricultural water systems, thus finding their way into various food commodities. The presence of these pollutants and contaminants in food and the environment has received considerable attention in terms of research in identifying, monitoring, technologies for their removal, and implementing legislation to address the issue [2,4,5,10,11,12,13,14]. 

The sheer volume in terms of the quantity and type of chemicals and materials that are currently used daily, as well as new chemicals and materials developed each year, further exacerbate the problems associated with pollutants and contaminants of emerging concern. For example, some reports estimate that the European Union has registered over 100,000 chemicals for industrial, commercial, and personnel use with ~400 million ton of them produced globally [15]. EUROSTAT publishes statistics on the amounts of chemicals produced in the EU that are hazardous to the environment and health, and over 70% of these are chemicals with important environmental concern [16].

Due to the prevalence and harmful effects of these pollutants to humans, animals, and the environment, it is critical to regularly monitor their levels in food and the environment in order to adopt appropriate mitigation interventions to limit their occurrence and associated deleterious effects. In this regard, analysis plays a vital role, thus the constant need for improved and efficient analytical methods. Conventional methods of extraction and analysis of these pollutants are often expensive, inefficient, tedious, and utilize large volumes of harmful organic solvents. Hence, there is need for greener, cheaper, quicker, and more efficient approaches. Magnetic nanomaterials (MNMs) seem very promising in this regard and are, thus, proposed for the extraction of these pollutants for analysis. This review describes the extraction of these pollutants using MNMs, with a focus on mycotoxins, pesticides, and pharmaceuticals. The design of these extraction materials and their recent applications in different scientific fields are also discussed.

## 2. Occurrence and Significance of Pollutants in Food Commodities

Access to safe and quality food with adequate nutritional value is a priority for every food consumer; however, along the food production chain, most foods often become contaminated by pollutants, such as pesticides, mycotoxins, and pharmaceuticals, before reaching the consumers. Contamination of these pollutants begins on the farm, and continues during pre- and postharvest, as well as during storage, making it a global challenge, particularly due to their various adverse health effects in humans and animals [17]. In this regard, various food safety authorities have introduced regulatory guidelines to limit their exposure in consumers [18]. The United States’ Food and Drug Administration (USFDA), and Environmental Protection Agency/United State Department of Agriculture (USEPA/USDA), together with the Joint FAO/WHO Expert Committee on Food Additives (JECFA), European Union (EU), and other regulatory agencies, have set up these guidelines and maximum limits [19,20], as shown in Table 1.

Mycotoxins are among the naturally occurring environmental pollutants of concern in food and feed. These are toxic biochemical compounds synthesized during the metabolic activities of mycotoxigenic fungal species. Exposure to them among humans occurs mainly via ingestion of mycotoxin contaminated food [21] with dermal, aerosol, and parental routes as other avenues. The significant mycotoxins, i.e., aflatoxins (AFs), ochratoxins (OTs), fumonisins (FUMs), trichothecenes (THs), and zearalenone (ZEN), either cause cancer, liver, and kidney damage and or compromise immune and nervous system function, which could be accompanied by vomiting and death in various animal species [22]. In Malawi, high occurrence of esophageal cancer has been reported to correlate with high consumption of maize contaminated with FUMs [23]. The occurrence of mycotoxins in grains (sorghum, maize, wheat, and their products) at concentrations exceeding the maximum limits recommended by the European Commission and the South African Government has been reported [24]. Elsewhere, Onyedum et al. [25] observed the occurrence of economically significant mycotoxins in maize, cassava flake (garri), millet, yam flour, sorghum, and rice from North-Central Nigeria. In that study, levels of AFs, the most potent group of naturally occurring carcinogens exceeded the maximum recommended limits established by the European Commission in some of the foodstuff. Despite several efforts to manage mycotoxin contamination in food, their prevalence in staple food products still persist indicating a critical food safety and public health concern, which must not be ignored.

Pesticides and pharmaceuticals on their part are other groups of environmental pollutants that, heretofore, remain elusive to most food and environmental safety interventions. Pesticides, in particular, are of much concern today as the world shifts towards mechanized farming—increasingly adopting inorganic agricultural practices with little regard to environmental safety and sustainability. Pesticides potentiate carcinogenic, gastrointestinal, reproductive, neurological, respiratory, and dermatological properties that manifest in humans, particularly in pregnant women, children, and older people [26]. Recent research data show high prevalence of pesticides in many food commodities intended for direct human and animal consumption, despite the various regulatory frameworks to control their applications in agriculture. For example, Galani et al. [27] investigated the occurrence of 99 pesticides in 72 samples of 12 agricultural products from Cameroon. The authors reported that at least 21 of these pesticides occurred at levels exceeding the EU maximum regulatory limits. The occurrence of pesticides in wheat samples was also reported in Algeria, and about 5% of samples contained levels above maximum residue limits [28]. The accumulation of organochlorine pesticides in fish species in South Africa was reported, and levels recovered in most samples were above those regulated by EU [29].

More recently, pharmaceuticals have gained increasing attention as environmental pollutants of concern due to their unintended effects on the environments, such as the conference of antimicrobial resistance in some pathogenic microorganisms by antibiotics. Indeed, many of these active ingredients from pharmaceutical products enter the environment as trace pollutants largely from their intended use in veterinary and human medical practices, personal care, and agriculture; nonetheless, their prevalence in recent times is concerning to public health, especially as more research reveals their potential hazards. According to Küster and Adler [30], approximately 10% of pharmaceutical products are of note regarding their potential environmental risks. Bommuraj et al. [31] reported the occurrence of three pharmaceuticals (ibuprofen, bezafibrate, and caffeine) in diary milk from Israel, while Goldstein et al. [32] reported the uptake of pharmaceutical by vegetables, such as tomatoes and cucumbers, irrigated with wastewater contaminated with pharmaceuticals. These pharmaceuticals causes tooth discoloration, vision problems, and allergic reactions in humans [33].

Indeed, the persistent occurrence of pollutants, such as mycotoxins, pesticides, and pharmaceuticals, in food, feed, water, and the environment is now of a global public health significance, as such requiring intensified efforts in order to control their prevalence. One of the ways to adequately manage and control these pollutants is by routine and efficient analysis to determine their incidence and levels to facilitate adoption of necessary combative interventions. In the analyses of contaminants, efficient extraction is a critical and often unavoidable step. Many of the conventional extraction methods for these pollutants are limited in one way or another, and there is a continuous quest for more effective analytical procedures to detect and quantify these pollutants. The application of nanomaterials as adsorbents for the extraction/removal of mycotoxins, pesticides, pharmaceutical, and other pollutants of concern in the environment has been identified as promising, effective, fast, and environmentally friendly. In the subsequent sections of this paper, we explored in detail the prospects and applications of MNMs for the extraction of various environmental contaminants, with focus on mycotoxins, pesticides, and pharmaceuticals in food commodities.

**Table 1:** The regulatory limits of pollutants in food commodities.

Food Commodities	Pollutants	Regulatory Limits	Reference
**Mycotoxins (μg/kg)**			
Processed cereal	OTA	3 (EU)	[34]
Unprocessed cereals (raw cereals and grains)		5 (EU)	[34]
Unprocessed cereals (wheat, barley and rye)		5 (JECFA)	[35]
Milk	AFM_1_	0.5 (JECFA)	[36]
Processed cereals (tree nuts, dried fruits, rice, peanuts, maize)	AFB_1_	2 (EU)	[37]
Cereals and processed cereal products, except corn and rice		2 (EU)	[34]
Corn and rice		5 (EU)	[34]
All foodstuffs		5 (SA)	[38]
All foodstuffs	Total AFs	10 (SA)	[38]
Ground nuts and processed cereals		4 (EU)	[37]
Cereals and processed cereal products, except corn and rice		4 (EU)	[34]
Food		20 (USFDA)	[34]
Corn and rice		10 (EU)	[34]
All foods except milk		20 (USA)	[38]
All foodstuffs	PAT	50 (SA)	[38]
Fruit nectar and fruit juices specifically fruit juice ingredi- ents in other beverages and apple juice		50 (EU)	[38]
Apple juice component of a food that contains apple juice as ingredient, apple juice, and apple juice concentrate		50 (USA)	[38]
Processed cereals/grains(flour, semolina, meals, flakes de- rived from barley, maize, and wheat)	DON	1000 (JECFA)	[35]
Processed wheat-based products		1000 (USFDA)	[34]
Processed grain (wheat, maize, and barley)		2000 (JECFA)	[35]
Unprocessed oat, durum wheat, and maize		1750 (EU)	[34,37]
Cereals ready for direct human consumption and other unprocessed cereals		1250 (EU)	[34,37]
Cereal flour (raw materials in food products)		750 (EU)	[34]
Processed grains (maize meal and flour)	FB_1_ and FB_2_	2000 (JECFA)	[35]
Unprocessed maize grain		4000 (JECFA)	[35]
Processed corn (corn meal, flour, and grits)		1000 (EU)	[34]
Unprocessed corn		4000 (EU)	[34]
Corn-based breakfast cereals and snacks		800 (EU)	[34]
Clean (processed) corn ready for mass production	Total FUMs	4000 (USFDA)	[34]
Clean corn for popcorn		3000 (USFDA)	[34]
Dry milled corn bran		4000 (USFDA)	[34]
Degermed dry milled corn products		2000–4000 (USFDA)	[34]
Unprocessed maize intended for wet milling		4000 (EU)	[37]
Processed maize (flour, grit, meal, and semolina)		1000 (EU)	[37]
Corn flour	ZEN	200 (EU)	[34]
Corn-based snacks and breakfast cereals		100 (EU)	[34]
Unprocessed cereals other than corn		100 (EU)	[34]
Unprocessed maize		350 (EU)	[34]
Cereal flour other than corn flour		75 (EU)	[34,37]
All product derived from unprocessed cereals intended for direct consumption (excluding processed corn-based foods)		50 (EU)	[34]
**Pesticides (mg/kg)**			
Sweet potatoes	Oxamyl	2.00 (USDA)	[39]
	Dichloran	10.00 (USDA)	[39]
	Parathion	0.05 (SA, EU)	[40]
	Deltamethrin	0.05 (SA), 0.01 (EU)	[40]
	Fludioxonil	10.00 (SA, EU, CODEX)	[40]
	Triazophos	0.05 (SA), 0.01 (EU)	[40]
	Azoxystrobin	0.03 (SA), 1.00 (EU)	[40]
Pineapples	Malathion	2.00 (SA), 0.02 (EU),1.00 (CODEX), 8.00 (USA)	[40]
	Oxamyl	0.05 (SA), 0.01 (EU), 1.00 (USA)	[40]
	Isozofos	0.05 (SA), 0.01 (EU)	[40]
	Fosety-al	20.00 (SA), 50.0 (EU)	[40]
	Thia-bendazole	10.00 (SA, USA), 0.01 (EU)	[40]
Tomatoes	Malathion	0.05 (SA), 0.02 (EU), 0.50 (CODEX)	[40]
	Oxamyl	0.02 (SA), 0.01 (EU, CODEX)	[40]
	Parathion	0.10 (SA), 0.05(EU), 1.00 (CODEX)	[40]
	Chlorpyrifos	0.50 (SA, CODEX), 0.30 (EU)	[40]
Mangoes	Chlorpyrifos	0.01 (SA, EU)	[40]
	Fenvalerate	0.05 (SA), 1.50 (EU, CODEX)	[40]
	Malathion	2.00 (SA), 0.02 (EU), 0.10 (USA)	[40]
	Deltamethrin	0.05 (SA), 0.01 (EU)	[40]
	Parathion	0.10 (SA), 0.05 (EU)	[40]
	Azoxystrobin	0.10 (SA), 0.70 (EU, CODEX), 2.00 (USA)	[40]
Strawberries	Azoxystrobin	5.00 (SA), 10.00 (EU, CODEX, USA) 0.50 (SA)	[40]
	Dimethoate	0.10 (SA), 0.50 (EU), 2.00 (CODEX)	[40]
	Difenoconazole	2.50 (USA)	[40]
	Captan	15.00 (SA, CODEX), 6.00 (EU), 20.00 (USA)	[40]
	Emamectin benzoate	0.04 (SA), 0.05 (EU)	[40]
Banana	Thiabendazole	3.00 (SA), 5.00 (CODEX)	[40]
	Triazophos	2.00 (SA)	[40]
	Chlorpyrifos	1.00 (SA), 2.00 (CODEX)	[40]
	Dichlorvos	0.10 (SA)	[40]
	Fenamiphos	0.05 (SA, CODEX)	[40]
Citrus	Azoxytrobin	0.50 (SA), 15.00 (EU, CODEX)	[40]
	Buprofezin	0.05 (SA), 1.00 (EU, CODEX)	[40]
	Azinphos-methyl	2.00 (SA), 0.05 (EU), 1.00 (CODEX)	[40]
	Chlorpyrifos	0.30 (SA), 1.50 (EU) 1.00 (CODEX)	[40]
	Dimethoate	2.00 (SA), 0.01 (EU), 5.00 (CODEX)	[40]
	Emamectin benzoate	0.01 (SA, EU)	[40]
Table grapes	Fenvalerate	0.05 (SA), 0.30 (EU)	[40]
	Fenthion	0.50 (SA), 0.01 (EU)	[40]
	Dimethoate	2.00 (SA), 0.02 (EU)	[40]
	Chlorpyrifos	0.01 (EU, USA), 0.50 (CODEX)	[40]
	Azoxystrobin	1.00 (SA), 3.00 (EU), 2.00 (CODEX, USA)	[40]
	Acephate	1.50 (SA, USA), 0.01 (EU)	[40]
	Deltamethrin	0.10 (SA), 0.20 (EU, CODEX)	[40]

Note: CODEX: FOA/WHO; OTA: Ochratoxin A; DON: deoxynivalenol; ZEN: Zearalenone; PAT: Patulin; Total AFs: sum of AFB_1_, AFB_2_, AFG_1_, and AFG_2_; Total FUMs: sum of Fumonisin B_1_, Fumonisin B_2_, and Fumonisin B_3_.

## 3. Nanomaterials

Nanomaterials have attracted tremendous attention in research and their industrial applications recently. They allow engineers, chemists, scientists, and physicians to work at cellular and molecular levels to produce efficient developments in the healthcare and life sciences, as well as other technological applications. The design and synthesis of nanostructure materials and nanoparticles for the removal of environmental contaminants ensure public health, environmental safety, and sustainability. They are significantly considered as great adsorbents because of their unique structure, extremely small size, functional features, and high surface area, which allow for their pre-concentration and efficient extraction of pollutants in food [41]. There are various types of nanomaterials, which include quantum dots, metal nanomaterials, carbon nanomaterial, and magnetic nanomaterials; in this review, emphasis is placed on MNMs.

### 3.1. Magnetic Nanomaterials (MNMs)

Magnetic nanomaterials (MNMs) are a category of nanoparticles with magnetic properties manipulated using magnetic fields. The utilization of their electrical, magnetic, chemical, and thermal properties in various analytical processes, such as extraction, pre-concentration, and clean-up (sample treatment), detection, and chromatographic techniques, helps in the development of new analytical strategies or the enhancement of traditional ones, with particular benefits of cost-effectiveness, improved extraction recoveries, selectivity, precision, and overall speed of extraction. The synthesis of these MNMs involves the use of various magnetic materials, which include iron (magnetite and maghemite), cobalt (Co), and nickel (Ni), with their various derivative compounds [42,43]. Among these materials, iron (Fe) oxides, such as Fe_2_O_3_ and Fe_3_O_4_, and their associated ferrite derivatives, such as CoFe_2_O_4_ and MnFe_2_O_4_, are the most widely used in the production of MNMs. This is due to the relative simplicity of their preparation, chemical stability, high magnetic moments, and compatibility with the various biological systems when compared with other metals/metallic alloys, such as FePt, Mn_3_O_4_, Ni, and Co [44].

Indeed, it is worth noting that, despite the reported efficiency and efficacy, applications of MNMs are usually targeted at compounds of interest. MNMs can be functionalized/modified with different chemical groups to achieve selective interaction or extraction of analytes of interest. This represents a major advantage of MNMs because their surface chemistry is usually tailored toward or favourable in the extraction of specific groups of compounds. Iron oxides are known to degrade organic compounds, decompose under acidic conditions, and easily react with O_2_ in air, and, as such, it is necessary to coat them with different protective layers of material, such as carbon nanomaterials, polymers, noble metals, or silica, that help improve their stability and also to introduce new functionalities and surface features [45]. The functionalization of the produced MNMs surface with different functional groups is simple and institutes various physicochemical properties on the materials in order to enhance their analytical applicabilities [46]. In the proceeding section of this review, we discuss different types of MNMs used for extraction.

#### 3.1.1. Maghemite (γ-Fe_2_O_3_) and Magnetite (Fe_3_O_4_)

Maghemite and magnetite are the two major groups of iron oxide, which occur naturally with attractive magnetic features that are promising for different applications. They are both soft ferrimagnetic materials with similar structures, except that, in maghemite, the Fe cation is in trivalent states, while magnetite has cations of Fe^2+^ and Fe^3+^ [47]. Magnetite and maghemite are common components of MNMs used in magnetic solid phase extraction (MSPE). Different methods for synthesis of these Fe oxides have been described in the literature, such as hydrothermal method, solvothermal method, flow injection synthesis, oxidation of magnetic nanomaterials, co-precipitation, flame spray pyrolysis, sol-gel synthesis, and thermal decomposition of organic precursors at high temperatures and microemulsion [47,48]. Maghemite is a good absorbent for the removal of heavy metals due to its low cost, efficiency, safety, ease of separation and recovery, ability to adsorb, large surface site, simple synthesis availability, and superparamagnetic features [49,50,51]. Tuutijärvi et al. [52] successfully used maghemite to extract arsenic (V) in water. The removal of chromium (VII) by maghemite from contaminated water was also reported [53]. Narimani-Sabegh and Noroozian [51] synthesized a maghemite-based nanoparticle from lepidocrocite through calcination and extracted antimony (Sb) from aqueous media (soft drinks, bottle alcohol, water, non-alcoholic beers, and orange drinks). Research conducted by Devatha and Shivani [54] reported a novel application of maghemite nanomaterial coupled with bacteria (*Bacillus substilis*) for the extraction of cadmium (II) ion in aqueous media with a recovery of 76.4%. Rajput et al. [55] used maghemite nanomaterial synthesized by flame spray pyrolysis for the extraction of copper (II) ion and lead (II) ion in water.

Magnetite is also a promising absorbent applied in various scientific fields, most especially in the extraction of environmental pollutants. For instance, Piovesan et al. [56] synthesized magnetite nanomaterial coated with chitosan (Fe_3_O_4_@CS) to extract parathion in food commodities (tomato, carrot, rice, orange, and lettuce). Similarly, González-Jartín et al. [57] also synthesized this nanomaterial for the removal of mycotoxins in liquid food products (beverages). In addition, the application of molecularly imprinted polymer magnetic nanomaterial (Fe_3_O_4_@EGDMA) for OTA removal in grape juice was reported by Turan and Şahin [58].

#### 3.1.2. Neodymium MNMs

Neodymium, a member of the rare-earth metal (Lanthanide group) has drawn attention in different studies due to its strong magnetic properties (perhaps one of the strongest known to man). It is presently utilized in the fabrication of permanent magnets that are applied in wind turbine, spindles for computer hard drives and electric motors [59]. Neodymium-based MNMs are synthesized using various methods, including gel combustion, hydrothermal method, solution co-precipitation, hydrogen plasma-metal reaction, and thermal decomposition and microemulsion [60]. Ahmad et al. [61] synthesized NdCl_3_ (neodymium (iii) chloride) embedded with OMC (ordered mesoporous carbon) for the removal of sunset yellow from aqueous solution. Similarly, the synthesis of Nd_2_O_3_ nanomaterial for extracting acid dye from aqueous media was reported [62]. In 2020, Chen and coworkers [63] prepared neodymium sesquioxide coated with graphene oxide nanocomposite and modified with glassy carbon electrode (GCE) for the determination of anti-cancer drug (raloxifene) in biological samples.

### 3.2. Magnetic Alloy Nanomaterials

A combination of different metallic compounds (i.e., alloys) have also been utilized to synthesize nanomaterials with unique physicochemical properties. Some of these magnetic nanoalloys are discussed.

#### 3.2.1. Iron-Nickel (FeNi) Alloy MNMs

Iron-nickel (FeNi) alloy MNMs exhibit attractive magnetic features and are studied widely for various applications. FeNi_3_ has large saturation magnetization, high thermal stability, and permeability [64]. These alloy MNMs are synthesized using various methods, such as hydrothermal reduction technique, sol-gel method, spray pyrolysis, coordinated coprecipitation, and chemical reduction method [65,66]. It has become popular among analytical scientists due to ease of synthesis and application, separation of magnetic nanomaterial easily by an external magnet, and efficiency in extracting a wide range of organic compounds under different extraction conditions [67]. Khodadadi et al. [68] successfully synthesized FeNi_3_@SiO_2_ magnetic nanomaterial catalyst for tetracycline degradation in a neutral environment. Farooghi and coworkers [69] used FeNi_3_@SiO_2_ magnetic nanomaterial for the removal of lead in aqueous solution. Research by Nasseh et al. [70] reported the extraction of metronidazole in neutral environment using FeNi_3_@SiO_2_ magnetic nanocomposite.

#### 3.2.2. Iron-Cobalt (FeCo) Alloy MNMs

Iron-cobalt (FeCo) alloy MNMs are soft ferromagnetic nanomaterials with special features, such as low coercivity, large saturation magnetization, high Curie temperature, high permeability, high anisotropy constant, and high anisotropy energy [71,72,73,74,75]. These magnetic alloy nanomaterials are utilized in different technological applications, including microwave devices, magnetic recording media and exchange-coupled nanocomposite magnets, hyperthermia, and drug delivery [76,77]. FeCo magnetic alloy nanomaterials are synthesized using techniques, such as mechanical ball milling, interfacial diffusion, chemical vapor deposition, chemical co-precipitation, pulse laser ablation deposition (PLAD), pyrolysis, and reductive decomposition of iron (iii) acetylacetonate and cobalt (ii) acetylacetonate [78,79,80].

#### 3.2.3. Iron-Platinum (FePt) Alloy MNMs

Iron-platinum (FePt) are hard MNMs that have recently gained popularity amongst researchers due to the outstanding magnetic features they exhibit, such as great magnetocaloric effects, strong chemical stability, large saturation magnetization, magnetic imaging, and high magneto-crystalline anisotropy [81,82,83]. FePt magnetic materials are broadly applied in the biomedical field, magnetic data storage, large permanent magnet performance, electrocatalysis, nanobiotechnology, magnetic recording media, and biological research [84,85,86,87]. These alloys are synthesized through heat treatment (thermal decomposition) of Fe(Co)_5_ (iron pentacarbonyl), polyol process (reduction of platinum acetylacetonate in a mixed surfactants and polyol), and reduction of Fe salts and Pt (acac)_2_ [81].

#### 3.2.4. Iron-Palladium (FePd) Alloy MNMs

Iron-palladium (FePd) alloy MNMs are hard MNMs because of their large magneto-crystalline anisotropy energy [88,89] synthesized using several methods, such as microwave irradiation, modification of chemical synthesis from iron-platinum synthesis procedure, modification of polyol procedure, and epitaxial growth electron beam deposition [90]. A Pd-rich iron-palladium (FePd) alloy material functions as an excellent hydrogen absorption kinetic and as a catalyst. These alloy MNMs are applied in ultrahigh magnetic recording media [91] and biomedicals [89]. Fe_70_Pd_30_ material being amongst the different systems has wide popularity among researchers due to its magnetic shape memory (MSM) and martensitic conversion effect. Different shapes of these nano-sized materials, such as nanorods, nanohelices, nanospheres, and nanotubes, have recently been reported [89]. 

### 3.3. Advantages and Limitations of MNMs

Magnetic nanomaterials exhibit advantageous features when compared to other non-MNMs. Such features include good dispersibility in solvents (facilitated by their size), high surface area, and possibility of functionalization/modification of their surface for improved specificity, range of sorbents, and/or adsorption efficiency, and ease of separation using an external magnet from complex matrices without the need for centrifugation or filtration steps [92,93]. The materials are also highly recyclable/reusable usually after appropriate rinsing, with analytes adsorbed by processes, such as sonication [92,94] use less amount of organic solvents and are often cost-effective [93,94,95].

Despite their many merits, MNMs have some limitations and one of which is their agglomeration and aggregation in media causing reduction in their intrinsic magnetic (superparamagnetic or ferromagnetic) features. This problem is solved by modification/functionalization of the MNM with different materials, such as silica oxide, graphene oxide, carbon nanotube, metal organic frameworks, molecularly imprinted polymers, covalent organic frameworks, aptamers, and immunoassay, amongst others [92,96,97]. Such action can increase the rate of transfer of electron due to their conductivity compared to unmodified MNMs [95]. For example, Xu et al. [19] modified MNM with carbon nanotubes for extracting pollutants in egg. The modification of MNM with molecularly imprinted polymer for the analysis of thiamethoxam and thiacloprid in honey was also reported [98]. Other limitations of MNMs are that bare MNM can easily be oxidized and form hydrated oxides in acidic atmospheres or, when exposed to air [92], the thermally unstable and stationary phase components of the MNM can completely or partly degrade during the desorption process at high temperature, which leads to reduction in precision and accuracy of the analysis. In fact, at pH < 4, the degradation of any magnetite present can take place allowing the formation of chelates between the free ions and the target analytes. Alternatively, magnetite particles acquire a negative charge at pH > 9 due to the binding of OH groups causing electrostatic repulsion between the adsorbent and anionic forms of target analytes [94].

In addition, due to the size of the nano adsorbents, they can be difficult to separate from complex matrices when compared to larger particles, particularly when the sorbent is non-magnetic. Larger particles can easily be filtered and, if denser than the samples, can be centrifuged to separate the analyte from the sample. MNMs are advantageous in this regard, and, despite their nanosizes, they can easily be extracted from the sample using an external magnet. 

## 4. Extraction of Mycotoxins, Pesticides, and Pharmaceuticals

Extraction is the major step involved in analyzing these pollutants. Different conventional methods have been used to pre-concentrate and extract these pollutants from environmental samples and food commodities before quantification using GC-MS and LC-MS. The various steps used for the extraction processes are subsequently discussed.

### 4.1. Steps for Regular Sample Extraction

#### 4.1.1. Liquid-Liquid Extraction

**Figure 1:**
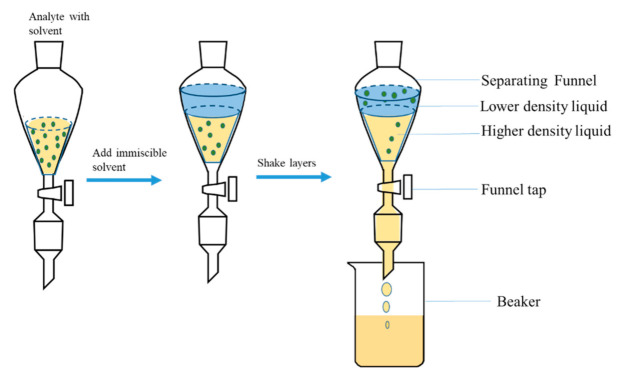
Diagrammatic illustration of liquid-liquid extraction (adapted from Nichols [100]).

Liquid-Liquid extraction (LLE), also called solvent extraction, is a common technique used in extracting and purifying analytes for further analysis. It is one of the oldest extraction methods but still the most frequently used. This extraction approach is based on two immiscible solvents, the aqueous solvent and organic solvent. The solvent containing the analyte is placed in a funnel, and an immiscible solvent is added, forming two layers which are shaken together [99,100]. The analyte then migrates from the initial solvent to the second solvent based on their relative solubility in the solvent [100] (Figure 1). However, this method uses large amounts of organic solvents (such as dichloromethane, sulfuric acid, potassium chloride, and acetonitrile), a laborious process, and it involves longer extraction time.

#### 4.1.2. Solid Phase Extraction

**Figure 2:**
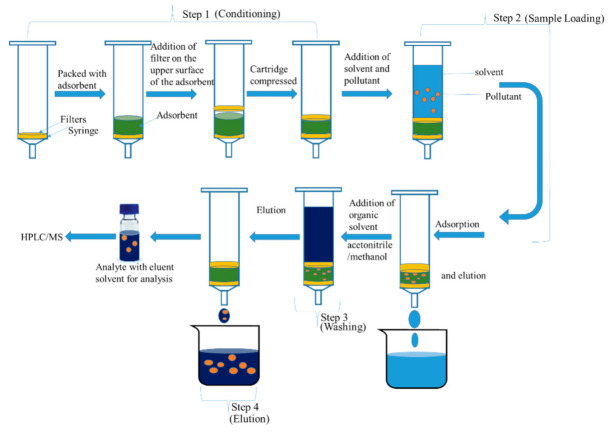
Diagrammatic steps of solid phase extraction (adapted from Badawy et al. [101]).

Solid phase extraction (SPE) is a frequently used method for extracting pollutants in aqueous samples. It basically involves four steps. The first step involves the use of cartridge filled with various types of particles with adsorption and adsorptive characteristics. It is loaded with sample in the second step, where the analytes of interest are retained. It is further washed with solvent to remove impurities (third step), and the analytes are eluted with a suitable solvent (fourth step) and kept for analysis using HPLC/MS [101,102] (Figure 2). SPE is mostly used for pre-concentration and clean-up of extracts in emerging pollutant analysis [103]. This method usually eliminates the need for expensive and environmentally sensitive solvents. However, there are limitations with this technique, such as loss of a compound of interest when loading the sample onto the sorbent, which causes clogging of cartridges by the sample’s suspended matter with the possibility of obtaining low recoveries by sorbent interaction towards the analytes [104,105].

#### 4.1.3. Quick, Easy, Cheap, Effective, Rugged, and Safe Extraction

This extraction method is an inexpensive and fast method for extracting pollutants in food using acetonitrile followed by dispersive solid phase extraction (DSPE). It is an effective sample preparation technique involving two steps. The first step is salting out to facilitate the equilibrium between the organic phase and aqueous phase, and the second step is clean-up employed by DSPE [106,107,108,109]. This technique is included as part of green analytical procedure due to it being an environmentally and user-friendly method. It uses little solvent, generates less waste [107], and can easily be modified using solvents, such as ethyl acetate and methanol [105], and clean up followed by filtering, SPE, freezing out, performing extra dilution, or LLE [106]. In 2018, Fernande et al. [110] used modified QuEChERS to analyze organophosphorus pesticides in strawberries with recovery values of 72–115%, limit of detection (LOD) and limit of quantification (LOQ) values ranging 3.64–10.38 µg/kg recorded. Similarly, the modified QuEChERS method for analyzing organophosphate pesticides in fruits and vegetables and recovery values in the range of 76.89–110.3 µg/kg, together with LOD and LOQ values from 0.1–1.0 µg/kg and 0.5–5 µg/kg, respectively, were reported [111]. More detailed reviews on QuEChERS, and its use on the analysis of various pollutants in various food commodities, have been reported in the literature [107,108,109].

#### 4.1.4. Immunoaffinity Column Extraction

Immunoaffinity column extraction is an antibody-based separation technique that involves the use of a stationary phase that is made-up of an antibody or antibody related reagent linked to a chromatographic matrix or magnetic beads and exploit the selectivity and strong binding ability of the antibodies to their target [112]. For instance, the extraction of AFB_1_ requires loading the sample extract in the column. As the extract passes through the column, the target analyte (AFB_1_) is retained by the antibody in the column. The step is followed by washing to remove impurities, and the analyte is eluted using an elution solvent that disrupts the binding between the AFB_1_ and the antibody [113]. This method attains selective and efficient enrichment in only one step as compared with other extraction techniques, but the production of the antibodies is time consuming, expensive, and difficult. In addition, it causes long waiting times for sample analysis as there is possible congestion and limited flow rate in the column [106,114,115]. Ye et al. [116] used immunoaffinity magnetic beads for analysis of OTA in oil and cereals. The results reported recovery values of 86.3–95.4% and LOD and LOQ values of 0.24 µg/kg and 0.80 µg/kg, respectively. A magnetic-separation-based homogeneous immunosensor for DON from wheat and sauce samples was reported alongside the LOD and recovery values (0.5 and 3.0 ng/mL) and (78.7–88.5%), respectively, recorded [117].

#### 4.1.5. Magnetic Solid Phase Extraction—Dispersive Liquid-Liquid Microextraction (MSPE-DLLME)

DLLME is the latest development in liquid phase micro-extraction. It involves the rapid injection of a dispersive and an extraction solvent into an aqueous solvent forming a cloudy solution, which produce microdroplets of the extraction solvent dispersed in the sample solution. The role of the dispersive solvent is to ensure that there is miscibility between the extraction solvent and the sample solution. The formation of a cloudy solution is to allow the instant separation of the analyte from the sample solution into the extraction phase. The cloudy solution is then centrifuged, and the extraction solvent containing the analyte of interest is collected by a microsyringe for analysis [118,119,120]. This extraction method has its advantages, such as minimal volume of solvent use, low-cost, simplicity, and high-speed extraction, but it also has such a drawback as low extraction efficiency [121]. This method is recently combined with other analytical techniques, such as MSPE-DLLME, SPE-DLLME, vortex-assisted-DLLME, air-assisted-DLLME, and ultrasound-assisted-DLLME to obtain extracts that are cleaner, have higher pre-concentration factor, and have better LOD values [119]. Yuan et al. [122] used MSPE-DLLME for extraction of herbicide in food (millet, oatmeal, barley rice, and soy), with good recoveries with LOD and LOQ values being 0.19–0.80 ng/g and 0.61–2.66 ng/g, respectively. Similarly, the use of MSPE-DLLME for extraction of three tetracyclines in milk was also reported with good recovery values of 70.6–121.5% recorded, together with LOD and LOQ values, respectively, of 1.8–2.9 µg/L and 6.1–9.7 µg/L [123].

#### 4.1.6. Magnetic Nanomaterials for Analytical Extraction

**Figure 3:**
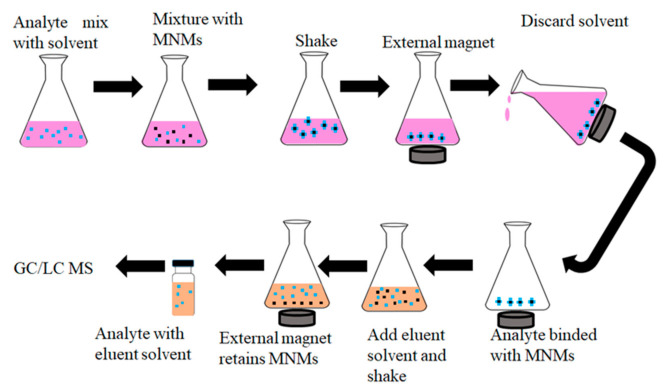
Diagrammatic illustration of magnetic solid phase extraction process for extraction of emerging pollutants.

The practical aspects of MSPE are made up of five steps, i.e., sample preparation, adsorption, extraction, desorption, and detection. MNMs have been used for the removal of various chemical compounds in different matrices. Magnetic solid phase extraction has advantages over conventional methods in many ways, such as high enrichment factor, shorter extraction time, fast separation using an external magnet, and easy operation [124,125]. For example, using an adsorption method on MNMs, major and minor pollutants are removed, where less than 1 g of the MNM is introduced into aqueous samples containing the dissolved pollutants [126]. This process of adsorption promises to show faster extraction than most of the other methods. The MNM is analytically maintained as a limiting reactant, so that the rate of extraction of pollutants depends on the amount of MNM used. The sorbent and the analyte are made to interact effectively for a certain period of time, and then the sorbent material adsorbs the analyte on its surface. After this, it is separated from the solvent by means of an external magnet. This shows that both the adsorption and desorption processes employed are easier to achieve as compared to other methods. Other methods require filtration and centrifugation steps, but MNMs avoid such steps and use an external magnet. Subsequently, a suitable eluent is used to wash the analyte from the magnetic nanomaterial before analysis. This helps eliminate the use of a solid phase extraction column [124,127,128,129]. Below is an illustration in Figure 3. Dispersive solid phase extraction is one of the commonly used extraction processes for the application of magnetic nanomaterials. This technique has been applied in determining pesticides and other pollutants, including mycotoxins in food and environmental samples [110,130,131].

### 4.2. Analytical Characteristics and Efficiency Parameters of Magnetic Nanomaterials

#### 4.2.1. Recovery

Recovery is one of the most important analytical parameters for studies involving extraction. It evaluates the closeness of agreement between acceptable values, and the experimentally observed values. Accurate quantification of pollutants in food commodities is essential in order to assess the compliance of the contamination levels of the pollutants in the sample with respect to the legal limits [132]. The EU recommended recovery values for mycotoxin is between 60–130% [132,133], pesticides from 70–120% [134,135,136], and pharmaceuticals between 80–120% [137]. The CODEX recommends 80–110% recovery values for mycotoxin [35] and 70–120% for pesticides [138]. The AOAC recommends recovery values between 70–125% for mycotoxins [132,139], while USFDA recommends 80–110% for mycotoxins [140] and 70–120% for pharmaceuticals residues at 10 ppb level [141]. USDA recommends recovery value for pesticide between 50–150% [141]. 

In the analysis of these pollutants, analytical approaches that yield recovery values which fall outside the recommended ranges as stated above are considered inaccurate and inadequate, which has been a recurring challenge of most conventional methods. Studies have reported low recovery values of 23.25–48.11% for some of the pesticides analyzed using the SPE method by Badawy et al. [101]. Likewise, low recovery values of 40–80% were reported for extracting pharmaceuticals using SPE approach [142]. In literature, effective recovery of emerging pollutants using MSPE method has been reported. For example, Ma et al. [143] reported recovery values of 80.2–108.3% for a simple magnetic solid phase extraction coupled to high performance liquid chromatography for the analysis of four heterocyclic pesticides from water. Similarly, the use of magnetite coupled to reduced graphene oxide nanocomposite for the extraction of isocarbophos in various sample matrices were also reported, with recovery values ranging from 81.0–108.5% [144]. The low recovery values reported by the conventional method is one of the SPE limitations, which is sometimes due to the blockage of the SPE column [101].

#### 4.2.2. Matrix Effect

Often, during extraction experiments, components of a sample other than the analyte(s) are co-extracted alongside the analytes, and they frequently interfere with analytical signals, thus compromising result quality. Matrix effect can compromise the precision, selectivity, reproducibility, sensitivity, linearity, and accuracy of the performance of bioanalysis assays leading to erroneous quantification [145]. Yavuz and coworkers [146] synthesized magnetic nanomaterial coated with polydopamine (Fe_3_O_4_@PDA) for the magnetic dispersive solid phase extraction of copper from food products and reported that the method had a good matrix interference tolerance. Peng et al. [147] used magnetic dispersive solid phase extraction for the analysis of five bisphenol compounds and reported a significant decreased matrix effect after clean-up by the prepared material.

#### 4.2.3. Limit of Detection (LOD) and Limit of Quantitation (LOQ)

Limit of detection (LOD) and the limit of quantitation (LOQ) are two important performance characteristics in analytical method development and validation. In the scientific literature, there are some vigorous debates on the use of both LOD and LOQ, in terms of definitions, calculations, and applications [148,149]. An acceptable general definition for LOD includes the lowest amount of analyte in a sample that can be detected by the instrument but not necessarily quantified [148,149,150]. On the other hand, LOQ is the lowest concentration of an analyte in a sample that can be detected and quantified by the instrument with suitable accuracy and precision [148,149,150]. These parameters can be determined by using the signal to noise ratio of approximately ≥3 for LOD and ≥10 for LOQ. In the case of LOD, the use of several blank samples and the resulting standard deviation from the measurements can be considered for techniques, such as LC/MS, where, in some cases, a reliable S/N cannot be determined [149]. Similarly, the LOQ can be determined using the slope of the calibration curve and the standard deviation of the response in the low concentration range [149]. For more detailed information and reviews on determining LOD and LOQ, as well as critical aspects on the use of LOD and LOQ, the reader is referred to the references [148,149,150].

The use of MSPE for determining ammonium compounds in vegetable and fruit puree samples using Fe_3_O_4_@NH_2_@G2 (cynanuric chloride-imidazole dendrimer functionalized iron oxide nanoparticles) was conducted. The study reported LOD and LOQ values of 0.05–0.50 µg/kg and 0.20–2.00 µg/kg [151]. Li et al. [152] synthesized amphiphilic block copolymer-grafted with magnetic multi-walled carbon nanotubes for the analysis of mycotoxins and pesticides using modified the QuEChERS method. The result showed LOD values of 0.00015–1.3 µg/kg. In 2021, the determination of ZEN in corn oil using magnetic molecularly imprinted polymer (Fe_3_O_4_@PDA@MIPS) was conducted with LOD value of 0.68 ng/mL recorded [153]. Fu et al. [154], on the synthesis of MCNTs (magnetic carbon nanotubes) to extract sulfonamides from milk following MSPE, found LOD and LOQ values of 0.002–0.01 ng/mL and 0.01–0.03 ng/mL, respectively. Table 2 shows different extraction methods and respective LODs and LOQs of pollutants analyzed. From the literature reviewed herein, it is observed that MSPE and QuEChERS methods have lower LODs for these groups of analytes compared to the other extraction methods.

#### 4.2.4. Reusability

Reusability is a desirable quality of extraction adsorbents. The reusability of the MNMs or nanocomposite is obtained by washing the materials several times with suitable solvents and then drying the materials for reuse. These materials are reused for as many times as possible, but still, demonstrate no obvious changes in the analyte recoveries [186,187]. The stability of the magnetic nanocomposite (PANI-Fe_3_O_4_) was studied under optimized conditions by evaluating the change in the analyte recoveries through various sorption-elution cycles, and the results indicated that the material might be reused fifty times [188]. Zhou et al. [186] reported the stability of Fe@MgAl-LDH nanocomposite during the magnetic solid phase extraction process with good reusability. Similarly, Zhou et al. [187] made similar observations with this magnetic nanocomposite (Fe@SiO_2_@p (NIP AM-co-MAA), in addition to good absorbent ability, which demonstrate that the materials have wide applications in analyzing real samples. In addition, a 10-time reusability of Fe_3_O_4_@SiO_2_@GO@PEA (phenylethylamine) was reported for the extraction of pesticides [189]. In another study by Wanjeri et al. [190], Fe_3_O_4_@SiO_2_@GO-MWCNT was reported to be reused up to 5 times for similar pesticides extraction. Both studies showed that the magnetic nanomaterials could be reused without a significant loss of adsorption.

#### 4.2.5. Extraction Time

An important factor in an effective MSPE adsorption process is the extraction time, which ultimately affects analytical output. Often, to reach the adsorption equilibrium, adequate contact time between the adsorbent and analytes is required. Wang et al. [191] reported optimized extraction time of 5 min for the extraction to be carried out. The extraction of AFB_1_ and ZEN in wheat flour by magnetic nanocomposite (Fe_3_O_4_@MWCNTs-NH_2_) was reported, and optimal extraction time for the study was 25 min [174]. Markus et al. [175] reported 5 min as the optimum extraction time in the adsorption of polar organophosphorus pesticides in vegetables.

#### 4.2.6. Quantity of Extraction Material (Adsorbent Dosage)

An advantage of magnetic solid phase microextraction is the use of relatively little amounts of the adsorbent. This essentially contributes to lower costs and less waste generation. In the extraction of pesticides from grape and tomato, a maximum adsorbent dosage of 30 mg was achieved and used in the study [179]. Zhou et al. [187] investigated the effect of adsorbent amount (20–60 mg) on analytical performance and observed that recoveries remained constant at 40 mg. This showed that the analytes could be completely adsorbed by the adsorbent at 40 mg, which shows that a higher sensitivity was achieved when 40 mg of the nanocomposite were used in the experiment.

## 5. Applications of Magnetic Nanomaterials for Extraction of Pollutants in Food Commodities

In food analysis, magnetic nanomaterials are of great interest because of the unique features they exhibit, which include ease of separation with an external magnet, large unique surface site, and high charge transfer capacity [192]. The use of magnetic nanomaterials in food commodities is widely increasing and becoming of interest to researchers. They are modified with various functional compounds, chemicals, and groups, which include molecular imprinted polymers, ionic liquids, graphene, graphene oxides, multi-walled carbon nanotubes, and silica oxides, amongst others, to ease the extraction of pollutants from food samples. The applications of these various MNMs with their modifications are shown in Table 2.

## 6. MNMs for Non-Target Analysis

Non-target analysis refers to the rapid detection/quantification or screening of both known and unknown analytes in a matrix. It is a key way in identifying emerging pollutants or contaminants by screening the matrices without reference standards, and there are no limitations to the number of analytes that can be detected. This analysis uses high-resolution mass spectrometry (HRMS), specifically quadrupole, with time-of-flight hybridization and Orbitrap™ instruments [130]. The key features of this analysis is that a fingerprint known as a total ion chromatograph is obtained for each sample, which is used to compare with existing sample profiles to reveal deviations or helps in the identification of unknowns [193]. The raw data obtained from previous analysis can be used to re-look at analytes that were not of interest, or not known, search for new compounds, or re-sampling or re-analysis of stored samples.

In non-target analysis, different extraction sorbents are used [194], but our focus will be on MNMs sorbents. For example, Jin et al. [195] did non-target analysis in vegetables using hierarchial micro and mesosphere metal-organic framework coated with magnetic nanosphere (H-MOF@Fe_3_O_4_). Non-target analysis was conducted using magnetic nanocomposite based on cellulose (Fe_3_O_4_@Cellulose) and different metabolites of enniatins and beauvericins screened in paprika samples [130]. In another study, a non-target analysis of over 204 pesticides was carried out by developing a magnetic blade-spray tandem mass spectrometry assay (MBS-MS/MS) [196]. In addition, the identification of pesticides and phytochromes from vegetables using heteropore covalent organic framework coated with magnetic nanosphere was reported [197]. The studies highlight the potential application of MNMs for sample pretreatment for non-target analytes. In addition, since MNMs can have their surface chemistry tailored and altered in a relatively short period of time, MNMs can provide new and rapid methods in achieving high performance sample preparation, and identifying new emerging contaminants of concern in various matrices, especially in a very wide range of food commodities.

## 7. Conclusions/Future Trends

The applications of MNMs in food analysis is of great interest to the scientific community because of their chemical and physical properties, which give them an edge to other adsorbent materials, thus being of great benefit to food safety. They have commonly been used in the MSPE process because of the ease of separation and recovery of analytes by an external magnet and the possibility of reusing them several times. As reviewed herein, MNMs can easily be modified using different materials, including graphene, ionic liquids, carbon nanotubes, and polymers, amongst others, to increase their surface area, thus enhancing their extraction efficiency and enriching the trace level of the analytes targeted in complex food matrices. Their specific features in terms of extraction capacity enable them have high surface site, and charge transfer capacity coupled to their ease of modifications, drawing a huge number of their applications in extraction. These MNMs and their nanocomposites have shown great potential for extracting various pollutants and could be applicable for efficient analysis of emerging pollutants and other important chemical contaminants in food and the environment. Despite numerous achievements in extracting pollutants using MNMs and their alloys, there are still gaps on the use of some alloys (i.e., FeCo, FePt, and FePd) for extraction, although they have been utilized in such fields as biomedical, microwave absorption, catalysis, magnetic data storage, nanobiotechnology, and magnetic recording media. There are therefore knowledge gaps in the applicability of MNMs for extracting environmental pollutants (particularly emerging pollutants). Other areas of interest could be the use of modern synthetic pathways, such as environmentally friendly solvents (ethanol and water) and naturally-derived plant extracts to synthesize and/or modify MNMs.

## Figures and Tables

**Table 2:** Current applications of MNMs in extracting pollutants from food commodities.

Type of Nanomaterial	Type of Modification	Morphological Characteristics/Size	Extraction Technique	Pollutants	Matrix	Recovery	LOD(ng/mL)	LOD Method	LOQ(ng/mL)	LOQ Method	Analytical Techniques	Reference
Nanosensor	Apt-PLNPs@cDNA-Fe_3_O_4_	TEM ZGO:Mn 48 ± 5 nm × 12 ± 1 nm ZGGO:Cr 20 ± 1 nm NH_2_-Fe_3_O_4_ 25 ± 3 nm	MSPE	AFB_1_ and ZEN	Grains (corn, rice, oats, wheat, millet, and corn grit)	93.6–105.1%	0.00022–0.00029	3s	Not provided	Not provided	Spectrophotometer	[155]
Nanocellulose	Fe_3_O_4_@Cellulose	Not provided	DMSPE	ENNs and BEAUs	Spice (paprika)	89.5–97.7%	2.8–3.0	3s	9.5–9.9	10s	UHPLC-MS/MS	[130]
Magnetic nanomaterial	g-C_3_N_4_/Fe_3_O_4_ (Graphitic carbon nitride/Fe_3_O_4_)	Not provided	Modified QuEChERs	27 mycotoxins	Maize	77.81–115.21%	0.004–0.6226	Not provided	0.0014–2.0753	Not provided	UPLC-MS/MS	[156]
Magnetic nanomaterial	Polypyrrole magnetic microsphere	SEM 2.81 ± 0.25 µm	MSPE	Carbaryl, carbofuran, and methomyl	Fruits and vegetables	81.6–108.3%	0.00137–0.0101	3s	0.00457–0.0331	10s	HPLC-DAD	[157]
Molecular imprinted polymers	Magnetic molecularly-imprinted polymer nanoparticles	SEM 2 µm	MMIP	Thiamethoxam and thiacloprid	Light and dark honey	96.8–106.5%	0.045–0.070	3s	0.07–0.1	10s	UHPLC-MS/MS	[98]
Magnetic nanomaterial	HCP/Fe_3_O_4_ (hypercrosslinked polystyrene/Fe_3_O_4_)	Not provided	MSPE	Nitrofuran metabolites	Honey	91–102%	0.1–0.3	3s	0.3–1.0	10s	LC-MS/MS	[158]
Magnetic bead	Fe_3_O_4_@AMP&ZnCl_2_@McAbs	Not provided	Immunoaffinity column	DON, ZEN, HT2, and T-2	Corn flour, oats, and wheat flour	76.60–99.47%	2–5	Not provided	5–20	Not provided	LC-MS	[159]
Carbon nanotubes	PEG-MWCNTs- MNPs	TEM 200 nm	MSPE	Mycotoxins	Milk	81.8–106.4%	0.005–0.050	3s	0.015–0.150	10s	UHPLC-Q Extractive HRMs	[160]
Magnetic nanomaterial	OAcFe-MNPs (Oleic acid/Fe_3_O_4_)	Not provided	DMSPE	Fenazaquin	Almonds	91.2–109.2%	0.06	3s	0.21	10s	GC-MS	[161]
Magnetic nanomaterial	Fe_3_O_4_@CTS@Apt (Aptamer-functionalized chitosan magnetic nanoparticles)	SEM/TEM Fe_3_O_4_: 13.2 nm Fe_3_O_4_@CTS: 18.5 nm	Magnetic extraction and immunoaffinity chromatography extraction	OTA	cornmeal	91.3–99.1%	Not provided	Not provided	Not provided	Not provided	HPLC	[162]
Carbon nanotubes	Fe_3_O_4_@MWCNTs@copolymer	TEM/SEM Fe_3_O_4_: 20–30 nm Coated with polymer: 5 nm	QuEChERs	mycotoxins and pesticides	Grains	60–108%	0.0002–1.3	3s	Not provided	Not provided	HPLC-MS/MS	[152]
Immunomagnetic nanomaterial	Fe_3_O_4_@CTS	TEM 400 nm	Immunomagnetic extraction	ZEN	cornmeal	91.7–104.3%	Not provided	Not provided	Not provided	Not provided	HPLC	[115]
Magnetic nanomaterial	MGO@LaP (lanthanum phosphate nanoparticles doped on magnetic graphene oxide)	SEM LaP nanoparticles: 20 nm	MD-µ-SPE	Chlorpyrifos and hexaconazole	Fruits juices	78–120%	0.67–0.89	3s	2.22–2.94	10s	GC-ECD	[163]
Magnetic nanomaterial	COF(TpPa-1)@ Fe_3_O_4_	Not provided	MSPE	Fluoroquinolones	Milk	90.4–101.2%	0.05–0.20	3s	0.19–0.71	10s	HPLC	[164]
Magnetic nanomaterial	SiO_2_-TiO_2_-NH_2_@Fe_3_O_4_	TEM 20 nm	MSPE	Malathion, chlorpyrifos, hexaconazole, and atrazine	Green and roasted coffee beans	74–113%	1.33–1.43	3s	4.45–4.77	10s	GC-ECD	[165]
Carbon sheet	Fe_3_O_4_-Cs (magnetic carbon nanotubes)	FE-SEM 15 nm	MD-µ-SPE	Malathion, chlorpyrifos, and fenthion	Vegetables and environmental samples	70–81%	0.46–1	3s	2–5	10s	GC-IMS	[166]
Magnetic nanomaterial	Fe_3_O_4_@GO/Apt	Not provided	MSPE	Chloramphenicol	Honey and milk	80.5–105.0%	0.24	3s	0.79	10s	HPLC	[167]
Magnetic nanomaterial	3DPCMs (Macroporous magnetic 3D photonic crystal microspheres)	Not provided	Immunoaffinity column	AFB_1_, OTA, and ZEN	Corn, rice, wheat	70.01–100.12%	Not provided	Not provided	Not provided	Not provided	HPLC–FLD	[168]
Carbon nanotube	Fe_3_O_4_@MWCNTs	Not provided	Modified QuEChERS	Pesticides, mycotoxins, and veterinary drugs	Eggs	60.5–114.6%	Not provided	Not provided	0.1–17.3	10s	UPLC–MS/MS	[19]
Carbon nanotubes	Fe_3_O_4_@MWCNTs	TEM 100–200 nm	Modified QuEChERs	Mycotoxins	Grains	73.5–112.9%	0.0006–1.6337	Not provided	0.0021–5.4457	Not provided	UPLC-MS/MS	[169]
Metal organic framework	Fe_3_O_4_/MIL-101 (Cr) (magnetic metal-organic framework MIL-101(Cr))	Not provided	MSPE	Triazine	rice	79.3–116.7%	0.00108–0.0181	3s	0.0036–0.0602	10s	HPLC-MS/MS	[170]
Quantum dot	Fe_3_O_4_@ MPA-CdTe QDs (mercaptopropionic acid-capped CdTe quantum dots)	Not provided	MNP-based immunoassay	Alternariol monomethyl ether (AME)	Fruits (cherry, apple, and orange)	87.2–92.0%	0.0003	Not provided	Not provided	Not provided	HPLC-MS	[171]
Metal organic framework	M-IRMOF	TEM Fe_3_O_4_: 15 nm	MDSPE	Epoxiconazole, fenbuconazole, difenoconazole, thiabendazole, and pyraclostrobin	Lettuce vegetables	74.8–99.5%	0.21–1.0	3s	Not provided	Not provided	HPLC-MS/MS	[172]
Polymer coated magnetic nanomaterial	TPN@Fe_3_O_4_@GO	FE-SEM Fe_3_O_4_: 22–50 nm TPN@Fe_3_O_4_:26–70 nm	MSPE	Imidacloprid and 2,4-dichlorophenoxyacetic acid (2,4-D) pesticides	Tomato, cucumber, and water	91.2–102.4%	0.17	3s	0.5–5.0	10s	HPLC-UV	[173]
Carbon nanotubes	Fe_3_O_4_@MWCNTs-NH_2_	Not provided	MSPE	AFB_1_ and ZEN	Wheat flour	88.8–96.0%	0.15–0.24	3s	0.52–0.83	10s	HPLC	[174]
Magnetic nanomaterial	Fe_3_O_4_-SP/GO (magnetite-sporopollenin/graphene oxide)	Not provided	MSPE	Organophosphorus pesticides (phenthoate, dimethoate, and phosphamidon)	Vegetables (green long pepper, reddish tomato, cucumber, and green long beans)	81–120%	0.02–0.05	3s	0.10–0.17	10s	GC-µECD	[175]
Magnetic nanomaterial	Fe_3_O_4_@SiO_2_@GO-β-CD (β-cyclodextrin)	Not provided	MSPE-DLLME	Oxytetracycline, doxycycline, and tetracycline	Bovine milk	70.6–121.5%	1.8–2.9	3s	6.1–9.7	10s	HPLC-UV	[123]
Magnetic nanoparticles	Fe_3_O_4_@rGO@ β- CD	Not provided	MSPE	Organochlorine pesticides	Honey	78.8–116.2%	0.00052–0.00321	3s	0.00173–0.01072	10s	GC-ECD	[176]
Magnetic nanomaterial	Fe_3_O_4_@GO	TEM 10–15 nm	MSPE	Patulin	Apple juice	68.7–83.6%	2.3	3s	7.7	10s	HPLC-UV	[177]
Carbon nanotubes	Fe_3_O_4_@MWCNTs	Not provided	MSPE	ZEN and its derivatives	Maize	75.8–104.1%	0.03–0.04	3s	0.07–0.10	10s	UHPLC-MS/MS	[178]
Magnetic nanomaterial	MG@SiO_2_-TMSPED (magnetic graphene-based silica-N-[3-(trimethoxysilyl)propyl] ethylenediamine)	TEM 10–30 nm	MSPE	Pesticides	Tomatoes and grape	82–113%	0.23–0.30	3s	0.76–1.0	10s	GC-µECD	[179]
Magnetic nanomaterial	Fe_3_O_4_@PAS-C_18_ (3-(*N*,*N*-diethylamino)propyltrimethoxysilane	Not provided	MSPE	Pesticides	Rice	82.2–125%	0.24–2.05	3s	2.0–14.9	Not provided	LC-MS/MS	[180]
Magnetic nanomaterial	Fe_3_O_4_@pDA (poly(dopamine))	Not provided	m-μdSPE	Estrogenic mycotoxin	Yogurt and milk	70–120%	0.21–4.77	3s	0.98–19.40	10s	LC-MS	[181]
Magnetic nanomaterial	Fe_3_O_4_@GO-β cyclodextrin	TEM 10 ± 3 nm	MSPE	Tetracycline and doxycycline	Milk	92.1–105.0%	0.00018	3s	0.00056	10s	Voltammetry	[182]
Magnetic nanomaterial	Fe_3_O_4_@G-CNPrTEOS	Not provided	MSPE	Organophosphorus pesticides	Cow milk	82–94%	0.01–0.6	3s	0.05–1.9	10s	GC-µECD	[183]
Molecularly imprinted polymers	Fe_3_O_4_@SiO_2_-MIPs (dummy molecular imprinted polymers)	TEM Fe_3_O_4_: 200 nm md-MIP: 20 nm	MSPE	Aflatoxin B_1_, B_2_, G_1_, and G_2_	Corn and tea leaves	75.6–94.8%	0.05–0.1	3s	Not provided	Not provided	UHPLC-MS/MS	[184]
Magnetic nanomaterial	Fe_3_O_4_@MDN (tetraethyl orthosilicate (TEOS) and methacrylic acid-3-(trimethoxysilyl) propyl ester (MPS))	SEM/TEM500–600 nm	MSPE	Triazole pesticides	Honey	90.5–105.9%	1.1–3.2	3s	3.3–10.1	10s	HPLC-MS/MS	[185]

Note: ENNs and BEAUs: enniatins and beauvericins; Apt-PLNPs@cDNA-Fe_3_O_4_: aptamer-modified persistent luminescence nanoparticles coated with complementary DNA-modified magnetic nanoparticle; ZGO:Mn and ZGGO:Cr (two types of PLNPs); TPN@Fe_3_O_4_@GO: triazine-based polymeric network modified magnetic nanoparticles/graphene oxide nanocomposite; Fe_3_O_4_@AMP&ZnCl_2_@McAbs: immunomagnetic bead based on the metal–organic framework materials conjugated with monoclonal antibodies coated with Fe_3_O_4_; PEG-MWCNTs- MNPs: PEGylated multi-walled carbon nanotubes magnetic nanoparticles; M-IRMOF: magnetic amino-functionalized zinc metal-organic framework; MDSPE: magnetic dispersive solid phase extraction; MSPE-DLLME: magnetic solid phase extraction- dispersive liquid-liquid microextraction; MMIP: magnetic molecularly imprinted polymer; MD-µ-SPE: magnetic dispersive micro-solid phase extraction; GC-ECD: gas chromatography electron capture detection; GC-µECD: gas chromatography microelectron capture detection; UHPLC-MS/MS: ultra-high performance liquid chromatography mass spectrometry; UPLC-MS/MS: ultra-performance liquid chromatography mass spectrometry; HPLC-MS/MS: high performance liquid chromatography mass spectrometry; HPLC-FLD: high-performance liquid chromatography with fluorescence detector; LC-MS/MS: liquid chromatography mass spectrometry; TEM: transmission electron microscopy; SEM: scanning electron microscope; FE-SEM: field emission scanning electron microscope‘ md-MIPs: magnetic dummy molecularly imprinted polymers.

## Data Availability

Not Applicable.
